# Professor Caldwell

**Published:** 1841-05

**Authors:** 


### PROFESSOR CALDWELL.

Our colleague, Professor Caldwell, embarked for Europe, at Boston, on the 3d of April, in the steam ship Caledonia. He has gone thither, not merely to commune with the distinguished men of England and France, but commissioned by our Board of Managers to make purchase of books and apparatus for the Institute. The Professor will return in the month of October, in time to enter upon his official duties, on the first Monday of November. Meanwhile, from his well known activity, we may hope for several letters from him, with which to enrich and enliven the pages of our Journal. D.

